# Constructing an architecture-based cybersecurity solution for a system

**DOI:** 10.1016/j.mex.2023.102010

**Published:** 2023-01-09

**Authors:** Mora-Castro Alejandro, González-Herrera Andrés, Villalón-Fonseca Ricardo

**Affiliations:** CITIC – ECCI, Universidad de Costa Rica, San José, Costa Rica

**Keywords:** Information security architecture, Cybersecurity architecture, Information security methodology, Cybersecurity methodology, Risk management, Architecture-based cybersecurity methodology

## Abstract

Cybersecurity can be effectively managed with an architecture-based approach, composed with three viewpoints, namely system, security and process. Using models for describing a system and its security objectives enables a systemic and exhaustive risk management process. The architecture approach produces an integral set of security policies and controls that can be fully maintained during the entire system life-cycle. Furthermore, architecture models support automation and high scalability, thus providing an innovative way for constructing and maintaining the cybersecurity for very large systems or even for system of systems.

This work describes details, technical aspects, and examples for the risk management process of the architecture, including the establishment of the system representation, the security goals, going through risk identification and analysis, up to the policies and control definition. Some highlighting points of the methodology follow.

• System representation is simple because it focuses only on aspects relevant to security purposes.
• Security objectives behave as an end-to-end guidance of the security, for the whole system and also during its life-cycle.
• Risk management can be done with existing methods and standards, but additionally supported with the comprehensive capability provided by the system representation and the security objectives.

## Graphical abstract

**Figure:**
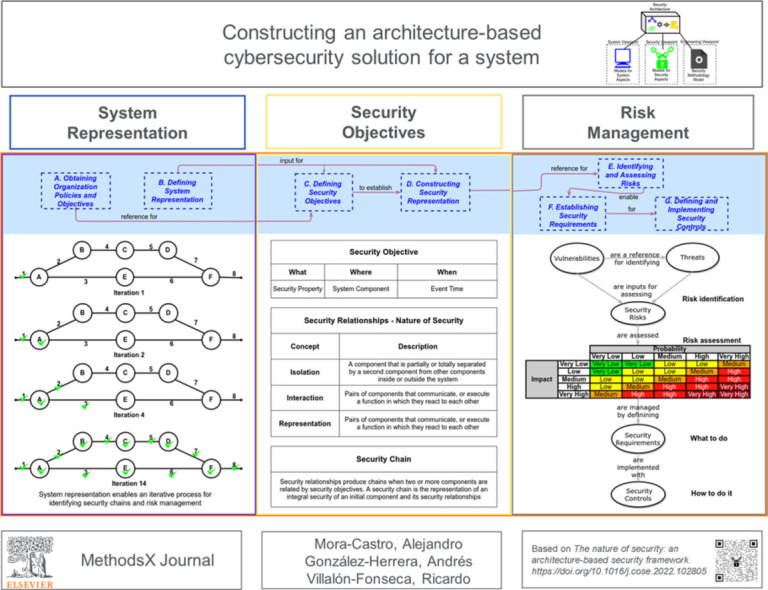
Constructing an architecture-based cybersecurity solution for a system (summarized)

Specifications table

**Table:** 

Subject Area:	Computer Science

More specific subject area:	*Cybersecurity Architecture*
Method name:	*Architecture-based cybersecurity methodology*
Name and reference of original method:	*The Nature of Security: A Conceptual Framework for Integral-Comprehensive modeling of IT Security and Cybersecurity*
Resource availability	https://doi.org/10.1016/j.cose.2022.102805

## Method details

Multidimensionality and complexity of cybersecurity problems can be managed with an architecture-based framework [3]. Models for describing a system and its security goals become a powerful tool for constructing the security requirements and controls for a system, with strong support for a systemic, comprehensive, and scalable security solution.

This work describes detailed aspects of the methodology proposed in [3] to construct a security solution, with the following steps:

A. Obtaining Organization Policies.
B. Defining the System Representation.
C. Defining Security Objectives.
D. Defining the Security Representation.
E. Identifying and Assessing Risks.
F. Establishing Security Requirements.
G. Defining and Implementing Security Controls.

**Fig. 1:**
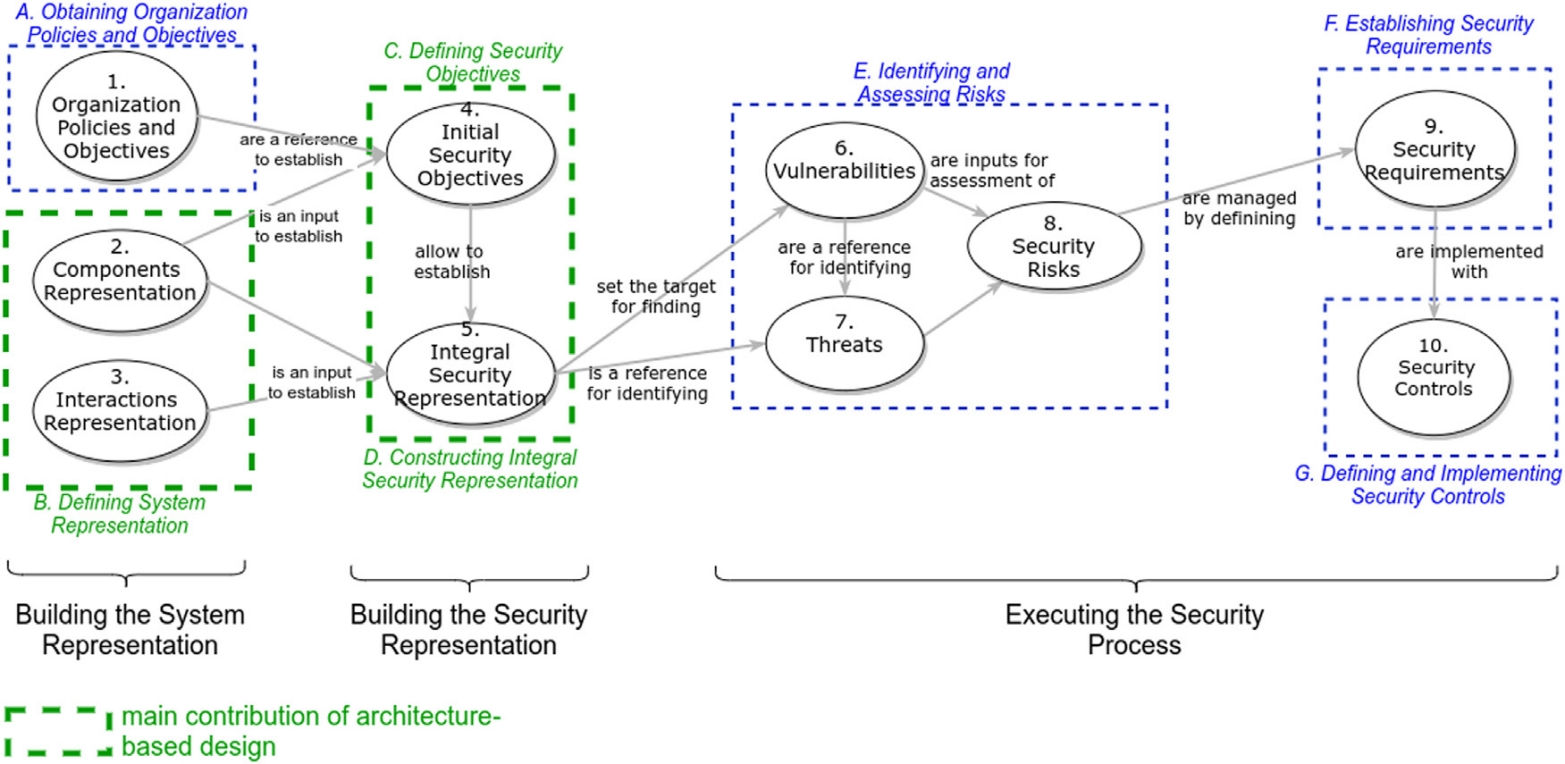
Architecture-based Cybersecurity Methodology. Source: [3].

This methodology allows to execute a security process for a system. The security process starts by collecting the business policy altogether with organization goals to define the high-level security objectives. The security objectives and the system representation are used to obtain a security representation for the risk assessment. The risk analysis produces security requirements to be enforced with security controls in the final step. Fig. 1 shows a conceptual map describing the methodology to be explained.

### Obtaining organization policies

Organizational policies and high-level goals are usually the guidelines for establishing the high-level IT security objectives of the entire organization and its IT systems. The methodology proposed in [3] does not propose a rigorous procedure for establishing initial high-level security objectives, but there are plenty of international standards, security guidelines and frameworks that can help to define the high-level security objectives for a system.

Starting with a simplified version of vision and mission statements up to an organization strategic direction, supported with international standards, can be used to propose the general goals for protecting sensitive data, software applications, infrastructure, and people related to the organization.

ISO/IEC 27001 standard provides clauses for defining security objectives that can be later used as the input for the methodology presented in this work. Similarly, the NIST Cybersecurity Framework (CSF) provides a set of five activities, which together are called the Framework Core, that provides a high-level strategic view for the organization. Additionally, the NIST CSF activities (namely Identify, Protect, Detect, Respond, Recover) provide relevant input not only for establishing the security objectives but also for describing the system target for the security.

Sometimes there are no high-level goals as the input for defining the security objectives, but there is some rationale or technical justification as a source for establishing the security objectives. As an example, consider the problem solved in [1], about implementing a security solution for digital signature software applications in Costa Rica, but excluding the non-repudiation property because that part of the solution was already solved separate. Table 1 describes the main elements of the initial objectives.

**Table 1:** High-level security objectives for a digital signature software app. Source: [1], English translated.

ID	Security Service/Property	Where (components)	When (event time)
1	Integrity	For information of the Application Software of the Application	During software application usage
2	Authentication	For users of the Application	During software application usage
3	Confidentiality	For information transferred to communicating components	While components Communicate

Integrity, confidentiality and authentication are common and usually required security services on software applications, but in particular for digital signature applications. Table 1 describes the goals in a generic way, where the target components are actually a large set of system components, including information and software components of the application. For propagating (detailing) the goals to more specific components, it is necessary construct a system representation describing the sub-components of the system, at a granularity level that is appropriate for a security process, as explained in the following section.

### Defining the system representation

A system representation allows to describe the specific components belonging to a system, but also those who participate on the security process, and consequently the scope of the security solution.

The system representation is carried out with two types of models or patterns, namely a whole-parts tree for describing the components and sub-components of the system, and one or more interaction diagrams (implemented with undirected graphs) for describing the security aspects associated to the functionality of the system. This section describes how to construct a whole-parts tree and the interaction diagrams for a system.

#### Creating the whole-parts tree

A whole-parts tree is a hierarchical data structure describing the components of a system, in a way that is appropriate for developing a security solution for the system. Before constructing a whole-parts tree, it is important to consider the following:

• The main purpose of the tree is to show what are the components of the system, including their subcomponents.
• No abstractions should be done when constructing the tree, with the exception of grouping as explained below. For example, it is not valid to include a non-existing software module that is not part of the system, it is not valid to reorganize and describe components in a way that does not represent their real structure/participation into the system, it is not valid to include components with functionality that does not belong to the system.
• The only valid abstraction for a whole-parts tree is grouping. Components can be coherently grouped, i.e. described as an actual group of the system. Groups allow to simplify the security process. For example, a security control can be applied to a group of components, such as a group of physical blade servers located inside a physical enclosure, or a security control can protect a group of virtual machines located as part of an IP network.
• The depth of the tree corresponds to the detail for describing the components and subcomponents of the system.But it will also impact the level of granularity of the security solution, and probably its effectiveness.

**Fig. 2:**
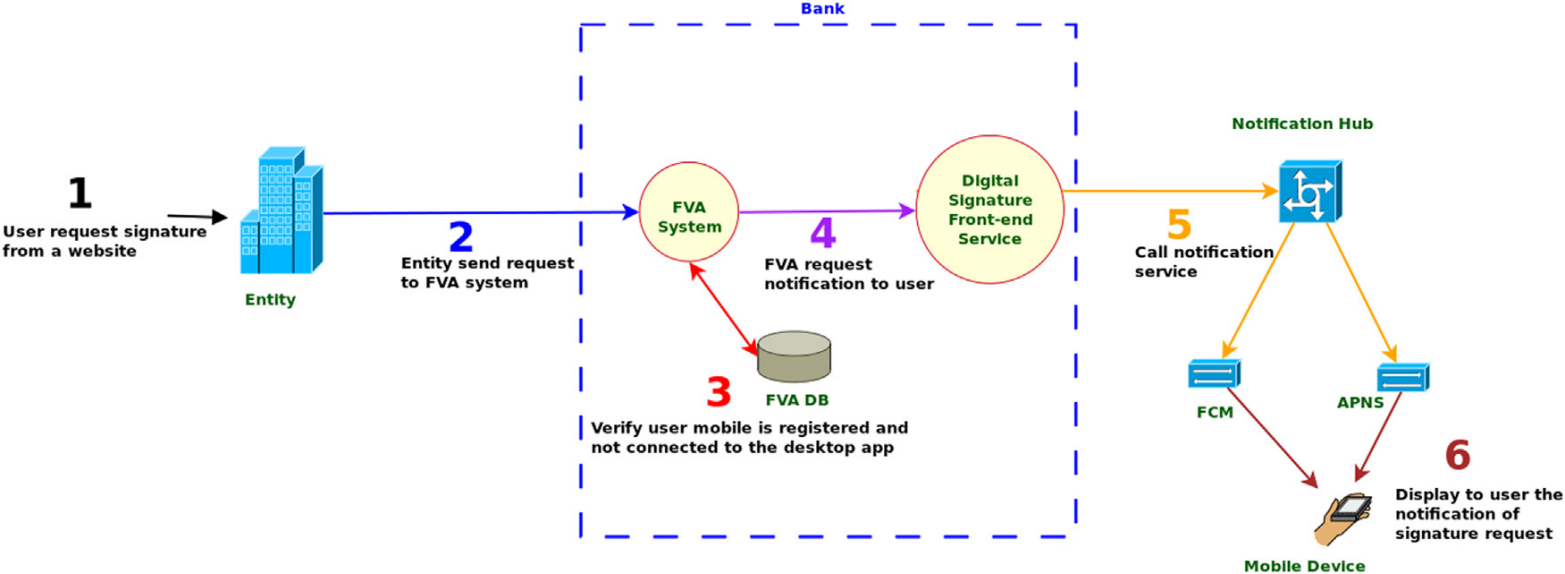
Digital Signature First Phase (Notification). Source: [1], English translated.

**Fig. 3:**
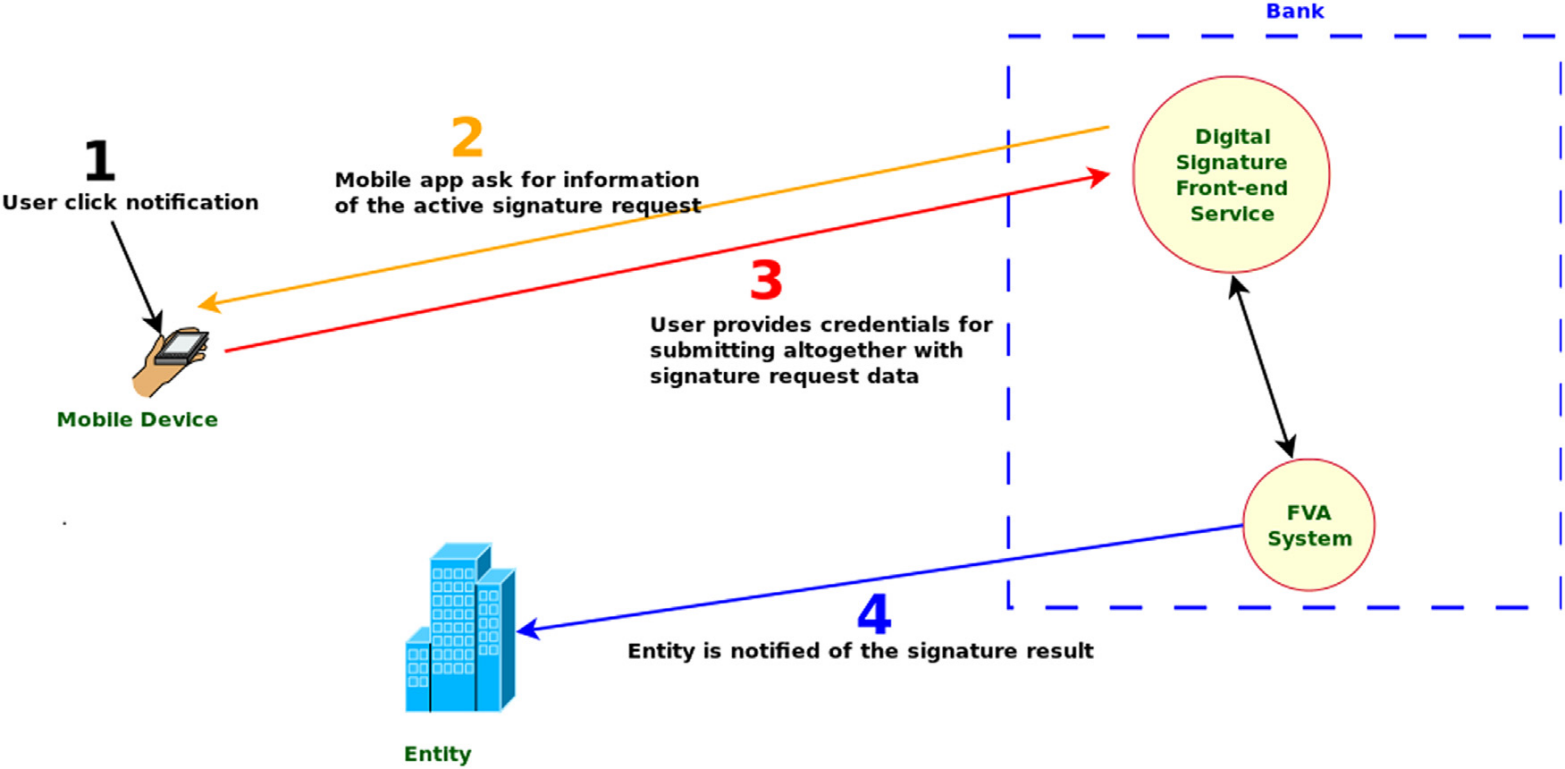
Digital Signature Second Phase (Signature). Source: [1], English translated.

Constructing a whole-parts tree can be done in several different ways, for example by initially creating high-level use case diagrams. Figs. 2 and 3 show a two-phase process, namely notification and signature, for requesting and digitally signing a document with a mobile device. The example corresponds to a digital signature software application of [1], that will be used for the rest of this work.

**Fig. 4:**
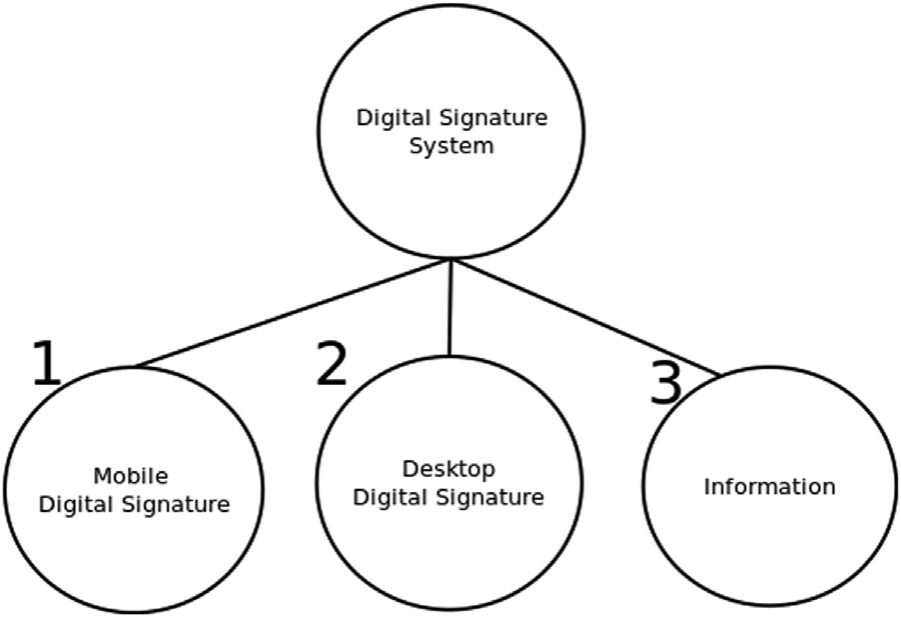
Upper levels of whole-parts tree for digital signature app. Source: [1], English translated.

**Fig. 5:**
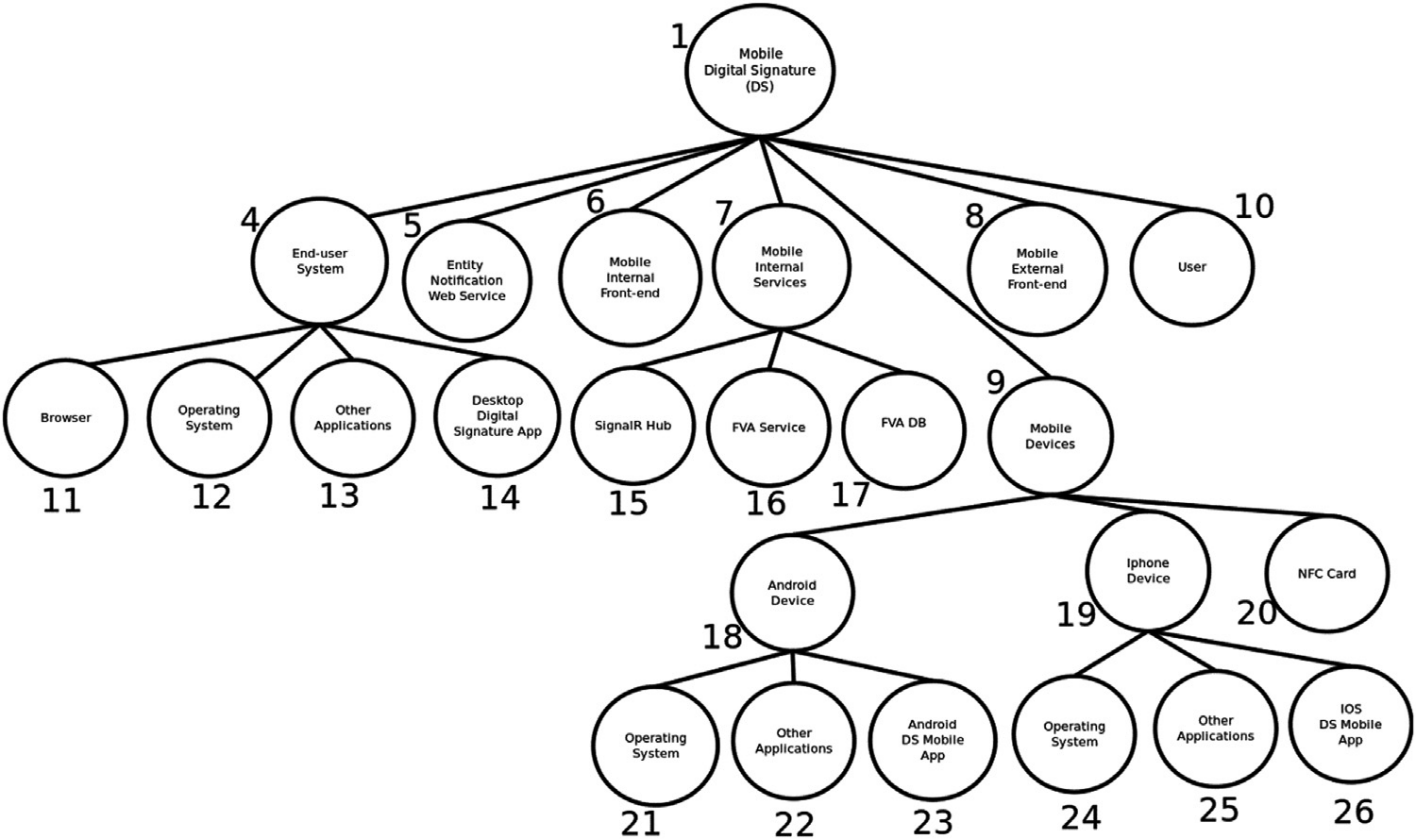
Mobile Digital Signature Sub-tree. Source: [1], English translated.

**Fig. 6:**
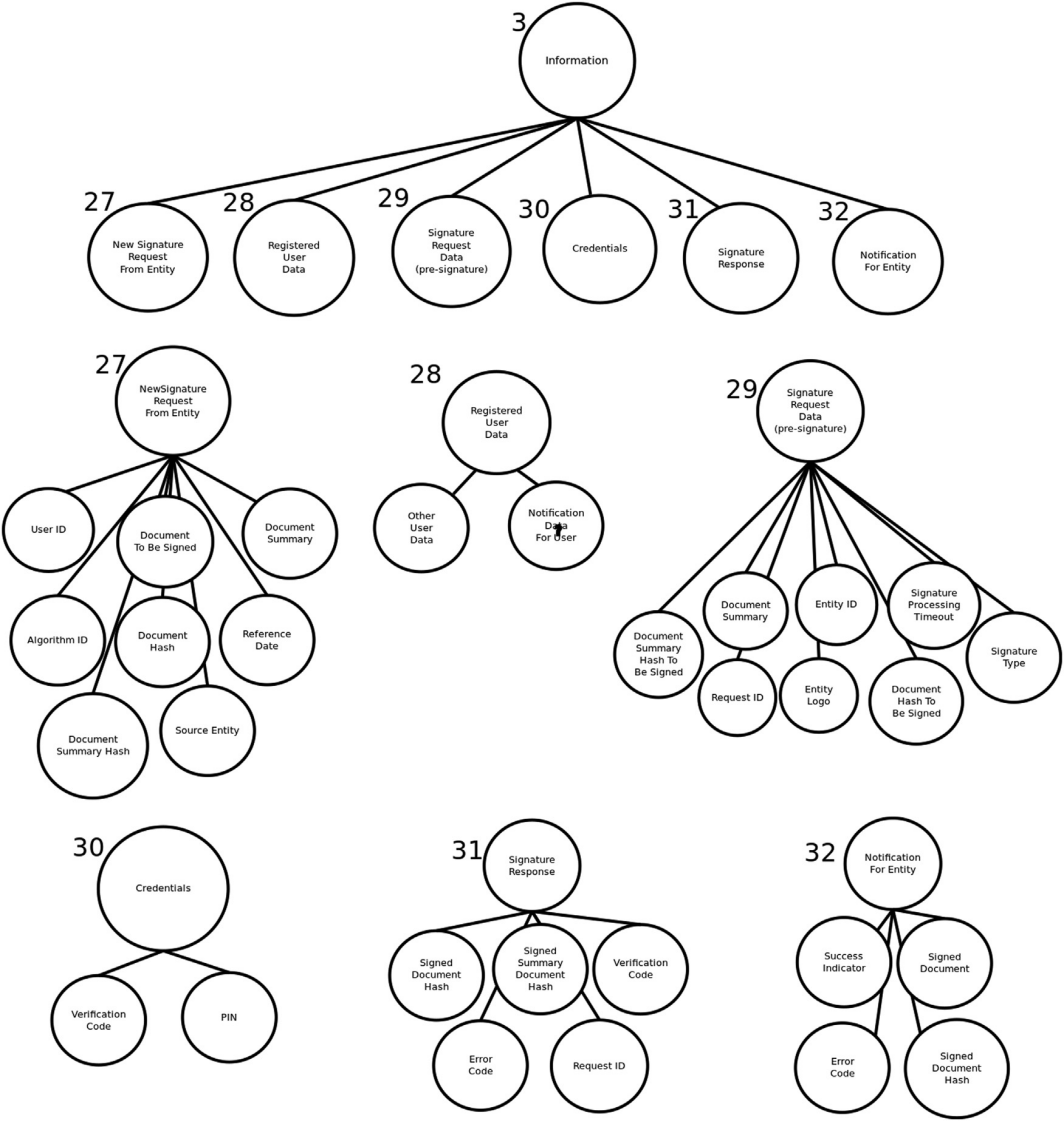
Information Sub-tree. Source: [1], English translated.

There are other useful approaches to obtain the input for constructing a whole-parts tree. For example, either the bottom-up or the top-down approach can be used because some components can be easily described/decomposed from general to specific or vice versa, and they can be later combined to create the entire tree, as shown on Fig. 4, Fig. 5, Fig. 6.

The first step when constructing the tree is to define a root node, for representing the entire system. Components below the root node, i.e. the second level of the tree, are usually large groups representing main category groups, such as information, software, physical infrastructure, or people. The second level could be also the main subsystems of the system. After that, additional tree-levels depend on the actual structure of the system.

The methodology for creating the whole-parts tree, as described in [3], provides a short but relevant list of considerations. They are reproduced here because of its importance to the process:

• “Gather a complete list of the system components. Including components of any category, such as IT hardware, network components, software, virtual resources like virtual machines or virtualized objects, information, and also people as long as they participate into the system. In general, any component or entity that is considered as an internal or composing part of the system should be included in the tree.
• Describe every component by its parts, as desired or required, by creating a corresponding subtree. For every component include a characterization of its properties, and aspects related to its structure. Include information about physical components that behave as separator, for example when a component is a container, or a boundary like a computer case.
• Check for correctness of the whole-part structures. There are common mistakes such as considering digital components (for example, operating systems and software applications) as containers. Software components or virtual resources are not containers but they interact with each other.
• Define groups of components as a tool for constructing the whole-parts tree. Groups are the only abstraction when building the tree. They allow to represent a set of components as one element, for applying security requirements to the group.
• Components like information, software and other digital resources may have a special treatment. They could be considered more than once. For example, a software application may be stored into file in a hard disk, but it could also be a running process inside a computer with CPU and memory. Then, representation of some components may be duplicated to satisfy different security requirements at different locations of the system. They may also be represented inside communication channels, when dealing with security of transferred components. Consider representation relationships for these components.”

#### Creating interaction diagrams

Interaction diagrams provide the functional aspects of the system, but only to satisfy the security perspective. Interactions can be established between components within the system, or with components outside the system. Interactions are established between pairs of components. A special type of interaction, called a communication channel, happens when two components interact but there is a third component moving between them, such as parameters with information running between two software functions.

**Fig. 7:**
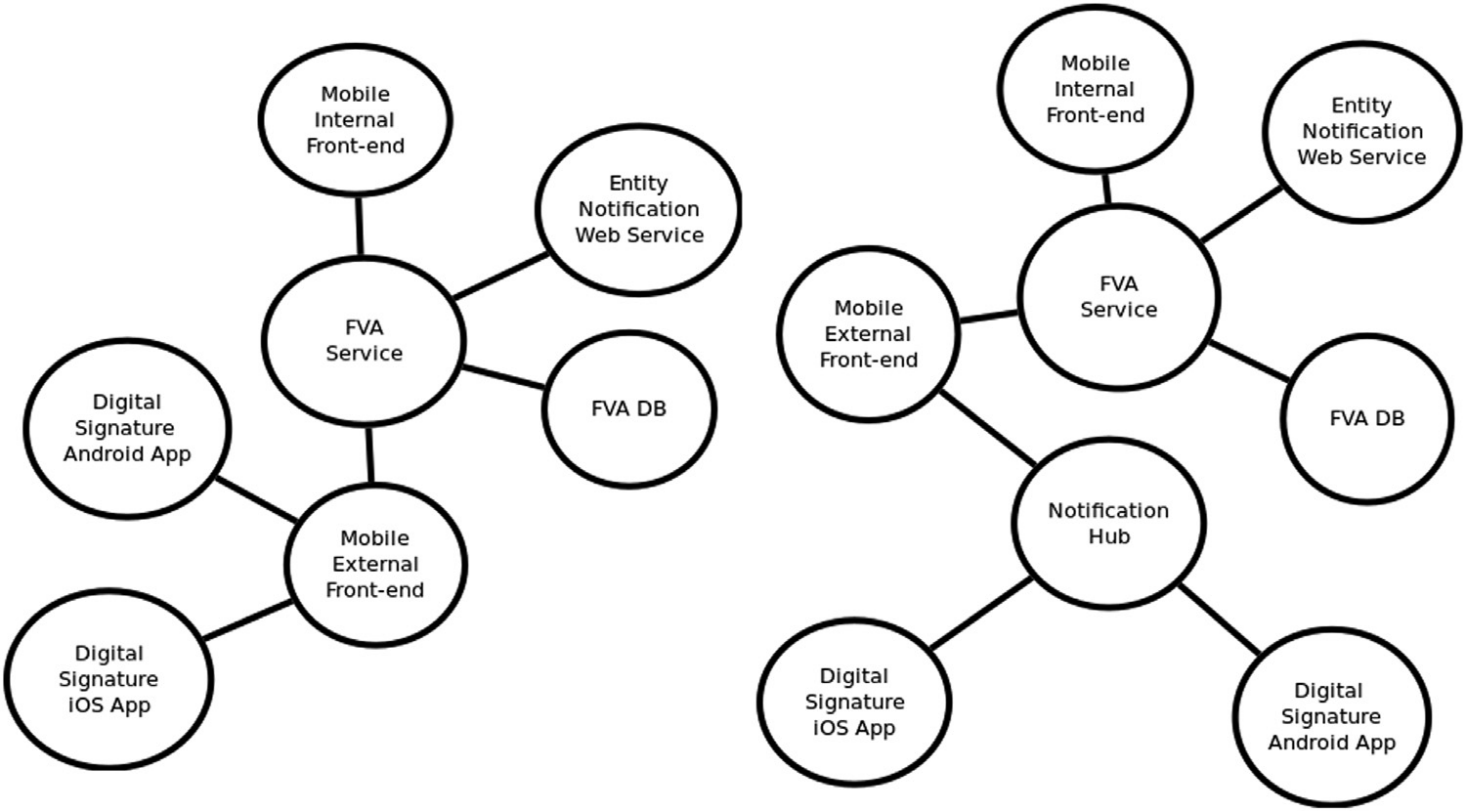
Interaction diagrams for the use cases of the digital signature app. Source: [1], English translated.

**Fig. 8:**
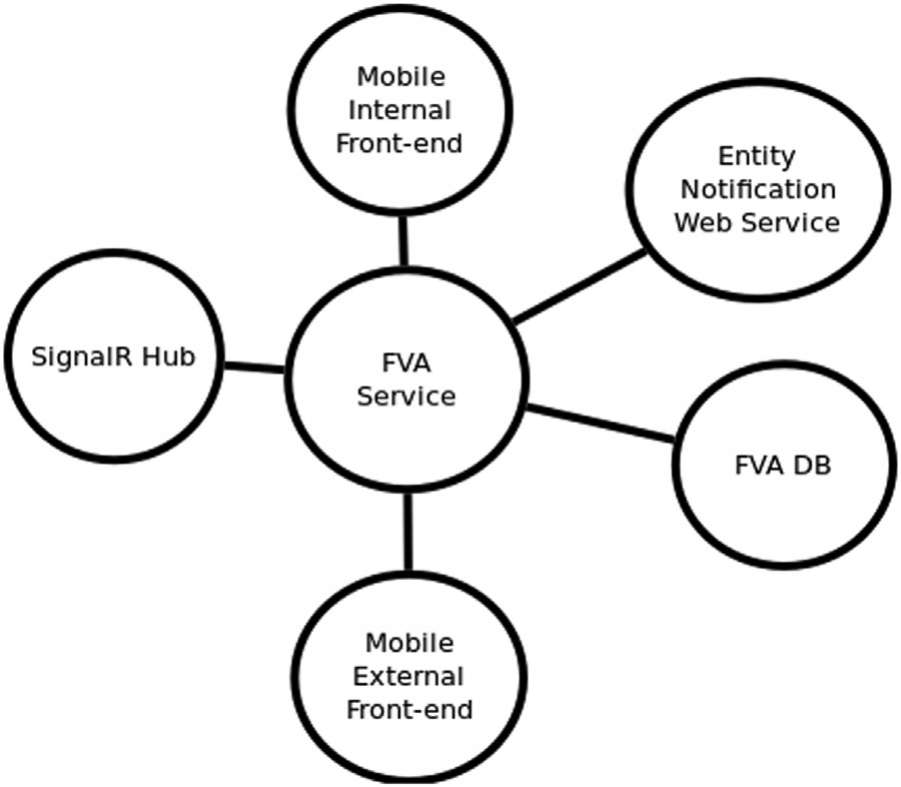
Interaction diagram for FVA Service, when not related to a use case. Source: [1], English translated.
• Interaction diagrams based on use cases require at least one diagram per each functional scenario, for comprising all the interacting components. Include enough diagrams to hopefully consider all interactions for all system components, or at least to include all components targeted by the security process.
• Interaction diagrams for specific components, i.e. with a star shape, do not require to identify complete uses cases, but direct interactions of the component with any other components of the system, independently of any identified use case. As seen on Fig. 8, this type of diagram is easy to implement because it only requires to iterate over all directly interacting components. But this approach is more complicated to follow and validate because it does not show sequences of interactions that are important to understand the system behaviour.

For constructing the interaction diagrams for a system, consider the following:

• There are two common ways for obtaining the interaction diagrams: a) from a use case of the system, like the use cases described in the system representation above; b) by indicating all direct interactions for each component. Fig. 7 shows interaction diagrams for the use cases of the digital signature software app. Fig. 8 shows the interaction diagram with only the interactions for the FVA Service of the same software app.

### Defining security objectives

Direct security objectives include not only the high-level security goals, like defined in the example of Table 1, but also more specific objectives so-called propagated direct objectives. To identify the propagated objectives, consider the following:

• Review the system for identifying components being relevant or sensible to the high-level security objectives, previously obtained from general policy guidelines and other inputs. Define additional security objectives for the identified components. Remember to indicate the security service or property, the component requiring that security, and the expected time frame for handling the security, i.e. before, during or after the security issues.
• Categories of components can be used to simplify identification of propagated objectives. For example, information components are usually the target of confidentiality or integrity requirements. Users and their digital representation, such as IDs, can be the target for authentication or authorization requirements.
• For reviewing the system components, you can browse the whole-parts tree, by scrolling down for all the node to associate propagated objectives to the relevant components or sub-components when applicable.

Table 2 shows a list of propagated direct objectives for the digital signature software application mentioned before.

**Table 2:** propagated security objectives (only for mobile use case). Source: [1], English translated.

ID	Propagated Direct Objective	Justification
OD [3][1]	Integrity for information components while processing the digital signature	By business objective 1
OD[27] [1]	Integrity for registered user data while processing the digital signature	Propagated OD [3][1]
OD[28] [1]	Integrity for new digital signature request from entity while processing the digital signature	Propagated OD [3][1]
OD[29] [1]	Integrity for digital signature request data (pre-signature) while processing the digital signature	Propagated OD [3][1]
OD[30] [1]	Integrity for credentials entity while processing the digital signature	Propagated OD [3][1]
OD[31] [1]	Integrity for digital signature response while processing the digital signature	Propagated OD [3][1]
OD[32] [1]	Integrity for entity notification while processing the digital signature	Propagated OD [3][1]
OD[33] [1]	Integrity for user notification data while processing the digital signature	Propagated OD [3][1]
OD[23] [1]	Integrity for digital signature Android app while processing the digital signature	By business objective 1
OD[26] [1]	Integrity for digital signature iOS app while processing the digital signature	By business objective 1
OD[23] [2]	Authentication for user on digital signature Android app while processing the digital signature	By business objective 2
OD[26] [2]	Authentication for user on digital signature iOS app while processing the digital signature	By business objective 2
OD[8] [3]	Confidentiality for information transferred to digital signature external front-end while processing the digital signature	By business objective 3
OD[15] [3]	Confidentiality for information transferred to SignalR Hub while processing the digital signature	By business objective 3
OD[5] [3]	Confidentiality for information transferred to entity notification web service while processing the digital signature	By business objective 3
OD[6] [3]	Confidentiality for information transferred to digital signature internal front-end while processing the digital signature	By business objective 3
OD[30] [3]	Confidentiality for credentials while processing the digital signature	By business objective 3

### Defining the security representation

A security representation becomes complete when the initial list of direct security objectives gets expanded, with additional indirect goals for securing other components, to sufficiently support the direct objectives. Indirect security objectives can be identified from existing security relationships between the components of the system, i.e. isolation, interaction, and representation. For example:

• A box protecting (isolating) the physical integrity of a hard disk may need additional protection for its own integrity.
• A software handling (interacting) a confidential piece of information may need additional integrity controls for its own code, to avoid a misbehaviour that would reveal the information.
• A digital ID (representing) for a user authenticated into a system may need additional integrity controls for itself to avoid an unwanted modification.

**Fig. 9:**
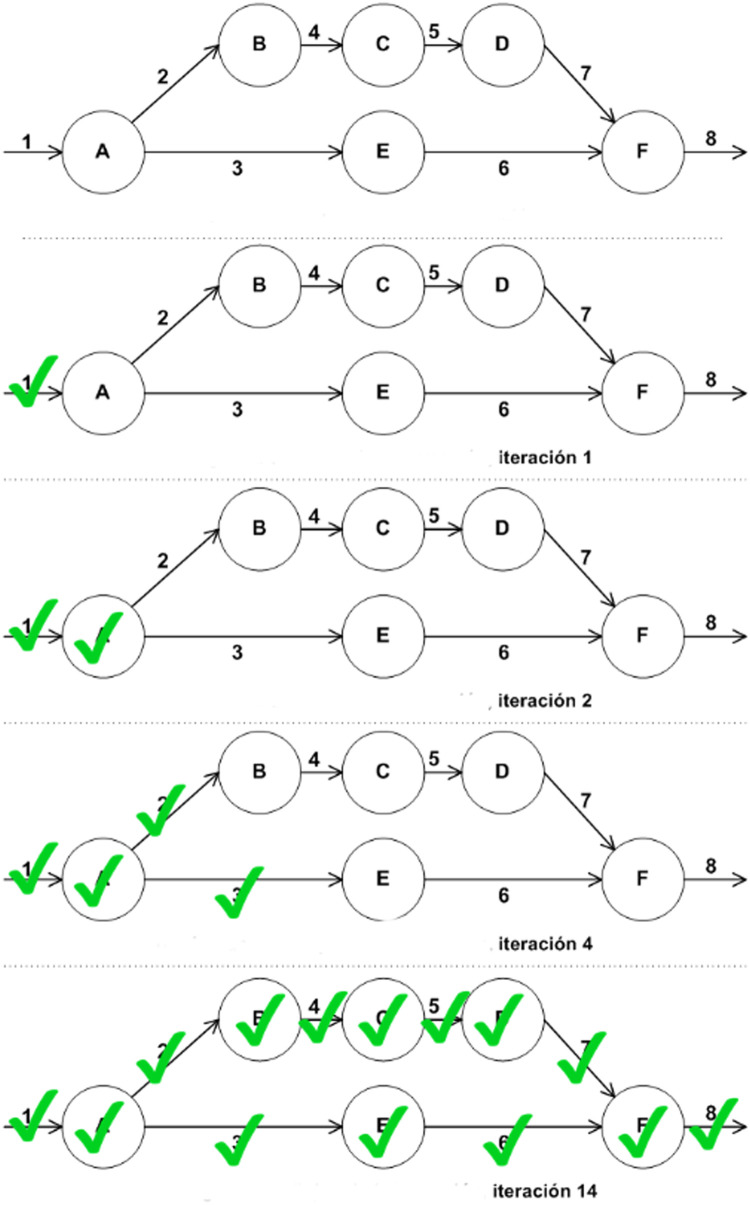
Iterative process for identifying security chains and also for risk assessment. Source: [2].

Sequences of security relationships can produce security chains to support the initial security objectives. This can be realized with an iterative process, as explained in [2] for the risk identification and assessment methodology, but the procedure can be also used for identifying security chains. Fig. 9 shows an example of an interaction diagram being iterated. For each node, and for each interaction (on this example considering interactions as communication channels) there are security objectives (associated with risks during the corresponding phase) to be considered.

In addition to interaction diagrams, components having representation relationships need to be considered for additional security objectives for the representative components.

### Identifying and assessing risks

Identifying and assessing risks is usually divided into several activities, and there are multiple international standards and guidelines for doing this task, as an example you can consider ISO/IEC 27005 for doing information security risk management. Here, we focus on the practical and systemic aspects that compliment any risk analysis methodology being considered, to achieve an integral, comprehensive and exhaustive risk analysis solution.

The entire risk process is guided by the same iterative procedure as shown in Fig. 9. All system components with security objectives (either direct or indirect) have to be visited to identify, assess and finally manage the associated risks.

#### Identifying risks

The risk process starts by identifying vulnerabilities and threats, related to the security objectives for the target components. Vulnerabilities and threats can be obtained from available online vulnerability databases, provider support systems, online communities, digital forums, as long as public or private security organizations. Vulnerabilities are to be associated with at least one potential threat to effectively identify a risk. A vulnerability not being associated with any threat may not be considered for a risk, but it may be monitored for changes. A similar reasoning follows for identified threats without any related vulnerability. Table 3 shows a categorization of vulnerabilities used for identifying vulnerability instances for the digital signatureapplication example.

**Table 3:** Sources of vulnerability by category. Source: [2], English translated.

Category	Source of Vulnerability
Technology	• Internet Browser • Application Frameworks • Operating System • Protocols
Hardware	• Hardware design errors • Deprecated or outdated hardware • Wrongly configured hardware
Software	• Incomplete or insufficient tests • Software errors • Software design errors • Software complexity • Corrupted software (with virus, malware, etc.)
Network	• Unprotected network communications • Open ports • Insecure network design • Too many privileges for users or resources • Unnecessary commands or scripts
IT Management	• Missing security updates • Insufficient incident management • Configuration errors

Identified risks determine the causes of a potential security problem, because they help to understand how, where and why the problem can happen, in the scope and boundaries of the system.

Additionally, Table 4 presents some threat examples. Having categories or an exhaustive list of threats is a challenging task, but also context dependant. Then, considering a risk identification approach based on vulnerability identification first, but also another iteration on threat identification first will probably provide an effective result when establishing the relevant risks for a system.

**Table 4:** Source threat examples by category. Source: [2], English translated.

Category	Threat source examples
Individuals	• Intruder • Staff / Collaborators • Trusted employee • Privileged employee
Groups	• Ad hoc • Recognized adversaries
Organizations	• Competitors • Providers • Partners • Customers

As an example, Table 5 shows fragment of resulting risks identified for the digital signature software application.

**Table 5:** Fragment of the resulting risk table. Source: [2], English translated.

ID	Source of Vulnerability	Source of Threat	Related Objective	Risk
1	Unprotected communications	Malicious attacker	OD[27][1]	Unprotected communications allow a malicious attacker to modify the electronic document to be signed or the other data of the signature request, while it is being transmitted from its original location to the "Mobile Internal Front-end " component.
2	Software defects	User	OD[27][1]	Defects in the "Mobile Internal Front-end" component allow the application to accept electronic documents from the user whose formats are not supported or are incorrect.
3	Software defects	Malicious attacker	OD[27][1]	Flaws in the “Mobile Internal Front-end” component allow the application to accept electronic documents containing hidden or malicious code from a malicious attacker.
4	Unprotected communications	Malicious attacker	OD[28][1] OD[8][3]	Unprotected communications allow a malicious attacker to modify or reveal registered user data while it is being transmitted from the “Mobile Device” component to the “Mobile External Front-end” component.

#### Assessing risks

Risks need to be assessed for determining if they should be mitigated. There are commonly used criteria for risk mitigation, such as the probability of occurrence, the impact for the system, or by using any other available methodology for risk evaluation. For the example digital signature software application, probability and impact were selected to assess the risks.

Probability of occurrence has aspects related to the vulnerability, and aspects related to the threat. These aspects are described in Tables 6 and 7, respectively, with a scale of values for the evaluation step.

**Table 6:** Aspects related to the source of vulnerability for determining risk probability. Source: [2], English translated.

Id	Aspect	Values
*H*	**Skill Level** What technical level is required by the threat source to find the vulnerability and exploit it?	• Requires a minimum level of technical skills = 9 • Requires a basic level of technical skills = 7 • Requires an intermediate level of technical skills = 5 • Requires an advanced level of technical skills = 3 • Requires an expert level of technical skills = 1 • Requires an omniscient level of technical skills = 0
*Ro*	**Reward** What is the magnitude of the incentive for the threat source to find the vulnerability and exploit it?	• Reward is maximum = 9 • Reward is great = 7 • Reward is moderate = 5 • Reward is small = 3 • Reward is insignificant = 1 • There is no reward = 0
*Ru*	**Resources** What is the amount of technological resources in space and time that the threat source needs to find the vulnerability and exploit it?	• Requires minimum resources = 9 • Requires few resources = 7 • Requires some resources = 5 • Requires enough resources = 3 • Requires many resources = 1 • Requires total resources = 0

**Table 7:** Aspects related to the source of threat for determining the risk probability. Source: [2], English translated.

Id	Aspect	Values
*D*	**Easy of Discovery** How easy is it to discover the vulnerability for a threat source?	• Immediate = 9 • Easy = 7 • Moderate = 5 • Hard = 3 • Very hard = 1 • Impossible = 0
*E*	**Easy of Exploitation** How easy is it to exploit the vulnerability for a threat source?	• Immediate = 9 • Easy = 7 • Moderate = 5 • Hard = 3 • Very hard = 1 • Impossible = 0

For calculating the risk probability an average formula over the aspects was selected, as shown in the following equation.

$$Probability\;of\;occurrence=\;\frac{H+Ro+Ru+D+E}{5}$$

Additionally, [2] mentions other ways of calculating the probability of occurrence, but they did not provide significantly better results for the risk analysis. They are described here only for future consideration and for having options to improve the risk evaluation process.

Suppose that *D* and *E* are proportional to *H* y *Ru*, as follow:

D∝HE∝H

D∝RuE∝Ru

Then, *D* and *E* can be written in terms of *H* y *Ru*, as follow:

$$D=\frac{H+R}{2}\;\;E=\frac{H+R}{2}$$

D+E=H+R

Thus, the probability of occurrence can be written as follow:

$$Probability\;of\;occurrence=\frac{Ro+2H+2Ru}{5}$$

Table 8 provides the mapping between values for the probability of occurrence and a qualitative description.

**Table 8:** Qualitative values associated to risk probability. Source: [2], English translated.

Range Value	Probability
[0, 1[	Very Low
[1, 3[	Low
[3, 5[	Medium
[5, 7[	High
[7, 9]	Very High

On the other hand, impact of risk has aspects related to the service, the business, financial and reputation, as described in Table 9.

**Table 9:** Aspects for determining the impact of the risk. Source: [2], English translated.

Id	Aspect	Values
*C*	**Consequences for interrupting an information security service** What is the level of consequences for interrupting an information security service as a result of the attack?	• Very high consequences = 9 • High consequences = 7 • Moderate consequences = 5 • Mild consequences = 3 • Very minor consequences = 1 • No consequences = 0
*A*	**Interrupting the business activity** What is the degree of interruption of the correct provision of the service as a result of the attack?	• Very high interrupt = 9 • High interrupt= 7 • Moderate interrupt = 5 • Low interrupt = 3 • Very low interrupt = 1 • No interrupt = 0
*E*	**Economic loss** What is the level of economic loss as a result of the attack?	• Incalculable economic loss = 9 • High economic loss = 7 • Moderate economic loss = 5 • Low economic loss = 3 • Very low economic loss = 1 • No economic loss = 0
*R*	**Reputation loss** What is the level of damage to the reputation as a result of the attack?	• Irreversible reputation loss = 9 • High reputation loss = 7 • Moderate reputation loss = 5 • Low reputation loss = 3 • Very low reputation loss = 1 • No reputation loss = 0

The risk impact was calculated as an average over the selected aspects, as shown in the following equation.

$$Risk\;Impact=\;\frac{C+A+E+R}{4}$$

Like with the probability, [2] mentions other ways of calculating the risk impact, but they did not provide significantly better results for the risk analysis. They are described here only for future consideration and for having options to improve the risk evaluation process.

Suppose that *A* is proportional to *C*, as follow:

A∝C

Then, the risk impact can be written as follow:

$$Risk\;impact=\frac{2C+E+R}{4}$$

Table 10 provides the mapping between values for the risk impact and a correspondent qualitative description.

**Table 10:** Qualitative values associated to risk impact. Source: [2], English translated.

Range of values	Impact
[0, 1[	Very Low
[1, 3[	Low
[3, 5[	Medium
[5, 7[	High
[7, 9]	Very high

As a final step, Table 11 shows an example of probability and impact altogether for defining the risk severity, and Table 12 shows a fragment of the resulting risk assessment for digital signature software application.

**Table 11:** Qualitative values for risk severity. Source: [2], English translated.

**Table 12:** Fragment of the resulting risk assessment table. Source: [2], English translated.

Risk ID	H	Ro	Ru	D	E	Prob.	C	A	E	R	Impact	Severity
1	3	9	5	3	3	**4.6**	9	9	7	7	**8**	**High**
2	3	3	9	5	5	**5**	3	3	3	3	**3**	**High**
3	1	9	3	1	1	**3**	7	7	7	7	**7**	**High**
4	5	5	5	3	3	**4.2**	3	7	3	3	**4**	**Medium**

### Establishing security requirements

When establishing security requirements, each security objective and its corresponding risks should be considered at least once. For the digital signature software application, risks with severity level of medium, high, o very high were selected for mitigation.

For establishing security requirement, every security objective should be considered altogether with the corresponding risks, to define the desired actions for their mitigation. It is usually common that multiple risks can be addressed with a single requirement.

Table 13 shows some security requirements established as a result of the selected risks for mitigation.

**Table 13:** Fragment of the resulting security requirements table. Source: [2], English translated.

Policy ID	Requirement or Policy	Mitigation
5	It must be validated that the digital signature request data does not contain hidden or malicious code	risks 3,8, 24, 28, 32,55,56
8	It must be validated that the data of the registered user does not contain hidden or malicious code	risks 4, 5
10	Registered user data must be protected while being transmitted over a network so that it cannot be modified by unauthorized users.	risks 4, 5
12	The data of the signature request must be protected, while it transits through local memory, so that it cannot be modified by unauthorized users.	risks 1, 6, 7,8, 9, 10
20	It must be validated that the signature request data is not delivered to unauthorized users.	risks 1, 6, 7,8, 9, 10,55,56
22	It must be validated that the format of the electronic document to be signed is supported by the digital certificate system and the application, and that it is also correct.	risk 2
23	It must be validated that the electronic document to be signed does not contain hidden or malicious code.	risks 3, 24, 28, 32
24	The electronic document to be signed, as well as its derivative representations (formatted electronic document, electronic document summary, encrypted electronic document summary, and signed electronic document), must be protected while being transmitted over a network, so that they cannot be modified by unauthorized users.	risks 1, 6, 7, 9, 10, 11, 12, 14, 15, 16, 17, 18,61,62,63
28	The electronic document to be signed must be protected, while it is stored within some component of the application, so that it cannot be modified by unauthorized users.	risks 1,2,3

### Defining and implementing security controls

Security controls are the mechanism for enforcing the security requirements. Every security requirement should be covered by at least one security control. Multiple requirements may be enforced with a single control, and multiple controls may be used to enforce a requirement in a multi-layered way.

Table 14 shows some security controls established to support the security requirements in our example.

**Table 14:** Fragment of the resulting security control table. Source: [2], English translated.

Control ID	Security Control	Policies
1	The data entered into the system entered by the user must be validated, verifying that each entry meets at least the following requirements: *When the input is text* • The entered characters must be valid, according to the corresponding allowed character set. • The length of the entered characters must be within the corresponding minimum and maximum limits. • If the input requires a specific format (such as a date, email address, phone number, etc.), the characters entered must conform to that format. • If the input is used as an argument in a create, read, update, or delete operation of records in a database, it must be done through parameterized statements (prepared statements), and not through the concatenation of character strings. • If the input is to be shown to the user later, during their interaction with the system, the corresponding escape rules must be applied depending on the language(s) used. *When the input is a file* • The file must be in a permitted format. • The file format must be correct. • The file size must not exceed a maximum allowed size. • The file must not store malicious content, such as viruses, malware, etc. • If the file will be stored on a server's file system, its name or location must not be the same as some configuration file depending on the type of server. For example, .htaccess in Apache, or Web.conf in IIS, amongst others.	5, 6, 7, 8, 22, 23
6	Document formats signed in simple format must be validated to correspond to one of the valid formats.	22, 23
7	The data must be transmitted over the network using a protocol that provides data encryption, with an effectiveness equal to or greater than that offered by TLS 1.2.	9,10,11, 14, 24, 25, 26
19	At least one authorization check must be satisfied, for delivery of the signed electronic document to be possible.	22, 23, 24, 27,28, 38

## Declaration of interests

The authors declare that they have no known competing financial interests or personal relationships that could have appeared to influence the work reported in this paper.

## Data Availability

No data was used for the research described in the article.
